# Triplet energy migration-based photon upconversion by amphiphilic molecular assemblies in aerated water†
†Electronic supplementary information (ESI) available: Experimental details, TTA-based UC mechanism, IR, absorption, emission and UC spectra, structure of **A2**, UC quantum yield. See DOI: 10.1039/c6sc01047d


**DOI:** 10.1039/c6sc01047d

**Published:** 2016-04-18

**Authors:** Hironori Kouno, Taku Ogawa, Shogo Amemori, Prasenjit Mahato, Nobuhiro Yanai, Nobuo Kimizuka

**Affiliations:** a Department of Chemistry and Biochemistry , Graduate School of Engineering , Center for Molecular Systems (CMS) , Kyushu University , 744 Moto-oka, Nishi-ku , Fukuoka 819-0395 , Japan . Email: yanai@mail.cstm.kyushu-u.ac.jp ; Email: n-kimi@mail.cstm.kyushu-u.ac.jp; b PRESTO , JST , Honcho 4-1-8, Kawaguchi , Saitama 332-0012 , Japan

## Abstract

A molecular self-assembly approach is developed to resolve an outstanding issue in triplet energy migration-based photon upconversion (TEM-UC), that is, air-stable TEM-UC in water. Amphiphilic cationic acceptor (emitter) molecules self-assemble in water *via* hydrophobic and hydrogen bonding interactions, with which anionic donor (sensitizer) molecules are integrated through electrostatic interactions. Triplet energy is quantitatively transferred from the excited donor to the acceptor, which is followed by effective triplet energy migration among the pre-organized acceptors. It leads to TTA and concomitant UC emission in water. The dense acceptor chromophore arrays with extended hydrogen bonding networks show efficient barrier properties against molecular oxygen, as demonstrated by the stable UC emission even in air-saturated water.

## Abstract 2

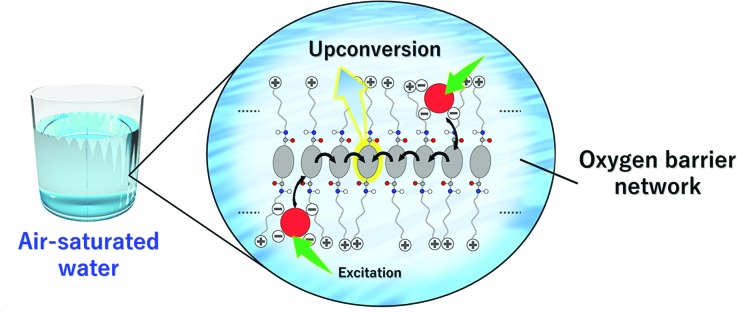
A molecular self-assembly approach is developed to resolve an outstanding issue in triplet energy migration-based photon upconversion (TEM-UC), that is, air-stable TEM-UC in water.

## Introduction

**Fig. 1:**
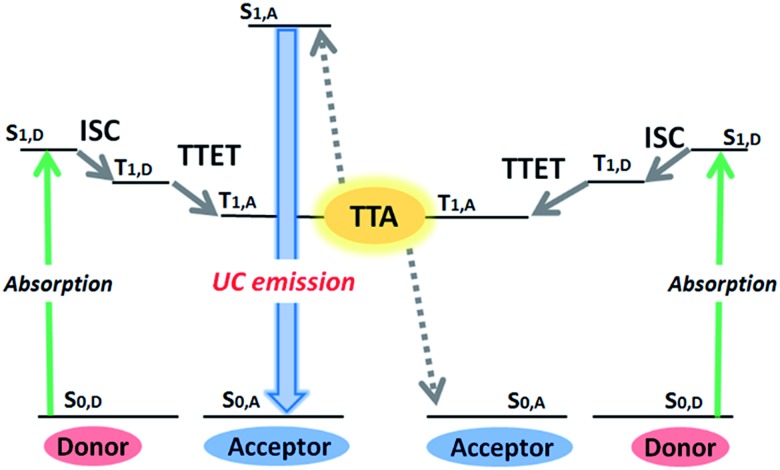
An outline of the TTA-UC process, showing the energy levels involved in the TTA-UC (S = singlet, T = triplet). The TTA-UC utilizes a pair of donor (sensitizer) with high intersystem crossing (ISC) efficiency and acceptor (emitter) with a high fluorescence quantum yield. Green and blue arrows indicate the absorption and emission processes, respectively. TTET: triplet–triplet energy transfer, TTA: triplet–triplet annihilation.

Photon upconversion (UC), converting lower-energy photons to higher-energy photons, has attracted considerable attention because of its wide variety of applications ranging from renewable energy production to bioimaging and photodynamic therapy.^1^–^5^ Conventional UC mechanisms such as two-photon absorption and multistep excitation of lanthanides, however, require strong excitation light intensity (>W cm^–2^), thus limiting their applications.^6^,^7^ On the other hand, triplet–triplet annihilation (TTA)-based UC with multi-component triplet donor (D, sensitizer) and acceptor (A, emitter) system can operate under a non-coherent, low intensity light source.^1^,^8^–^15^ As summarized in Fig. 1, this mechanism starts with the generation of donor triplets (T_1,D_) by intersystem crossing (ISC) from the photogenerated singlet state (S_1,D_), and the succeeding D-to-A triplet–triplet energy transfer (TTET) forms optically dark, metastable acceptor triplets (T_1,A_). The subsequent diffusion and collision of two excited acceptor triplets generate a higher energy excited singlet state (S_1,A_) through TTA, from which the upconverted delayed fluorescence is emitted. The TTET and TTA processes occur *via* an electron exchange mechanism (Dexter energy transfer), which requires the involved molecules to come close within the distance of 1 nm.

In spite of the growing demand for photocatalytic and biological applications, most studies on TTA-based UC involve investigations in deaerated organic solvents, and examples in air-saturated aqueous phase have been limited because of the massive quenching of excited triplet states by dissolved molecular oxygen. Previous reports have shown that this oxygen quenching can be suppressed by employing specific solid polymers or viscous liquids as matrices to reduce the oxygen concentration.^16^–^21^ For example, Kim and coworkers have reported photocatalytic reactions in aerated water by encapsulating UC chromophores in viscous hexadecane/polyisobutylene mixture.^17^,^19^,^21^ Another strategy is to utilize viscous matrices with singlet oxygen scavenging ability.^22^–^26^ Li *et al.* observed UC emission in aerated water by loading UC dyes in nanocapsules containing reductive linoic acid and oleic acid, and applied their nanomaterials to *in vivo* bioimaging.^22^ While these approaches allow for air-stable UC emission with reasonably high efficiency, the limited diffusion of large dye molecules in these viscous matrices is forecast to cause potential issues for further advancements.

An alternate strategy for aqueous UC is to take advantage of fast triplet energy migration (TEM) in condensed chromophore assemblies.^12^,^15^,^27^–^32^ In essence, TEM-UC is the chemistry challenge of how to pre-organize donor and acceptor molecules for effective donor-to-acceptor TTET and inter-acceptor TEM.^32^ TEM-UC works even in the absence of molecular diffusion, and therefore it has the potential to solve the inevitable issues associated with molecular-diffusion-based UC. While air-stable TEM-UC has recently been achieved in solvent-free liquids and self-assembled acceptors in organic media,^12^,^15^,^28^,^29^ oxygen-tolerant TEM-UC has not been realized in aqueous media.

**Fig. 2:**
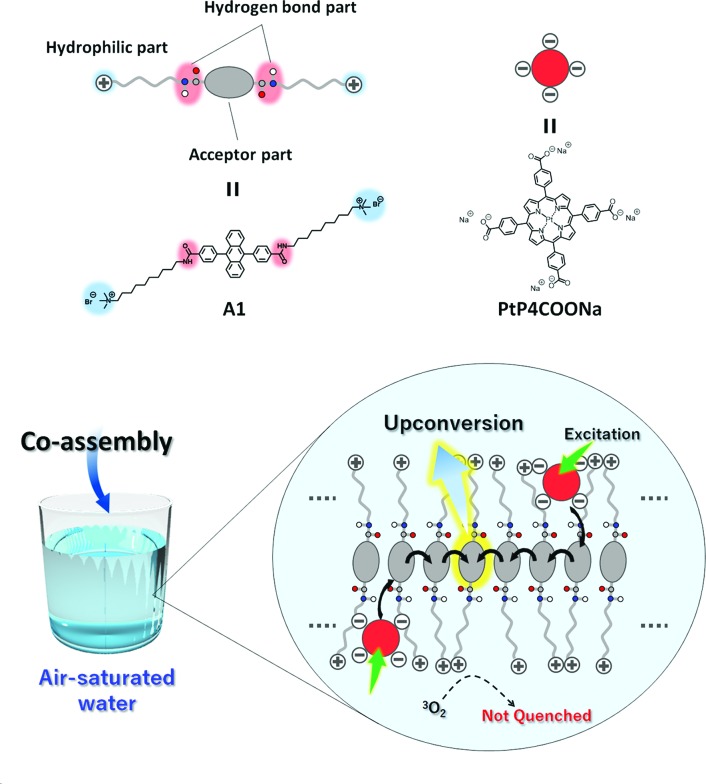
Schematic illustration of the aqueous TEM-UC system. Amphiphilic acceptor **A1** self-assembles in water to form monolayer membranes which are stabilized by hydrophobic and hydrogen bonding interactions. Anionic donor PtP4COONa co-assembles with the cationic acceptor membrane **A1** in water. Photoexcitation of the donor is followed by ISC, donor-to-acceptor TTET, TEM among the acceptor arrays, interacceptor TTA, and upconverted photoluminescence. Oxygen quenching is effectively avoided by the developed intermolecular hydrogen bonding networks in the acceptor monolayer membranes.

In this work, we show the first example of an air-stable aqueous TEM-UC system (Fig. 2). Ordered aqueous molecular self-assemblies such as bilayer membranes have been shown to give regular chromophore alignment and exert efficient singlet energy transfer characteristics.^33^,^34^ We made the assumption that such densely organized molecular assemblies with extended molecular networks such as hydrogen bonding may prevent the intrusion of molecular oxygen from the bulk water into the hydrophobic interior of the molecular self-assemblies.^35^

As a proof of concept, we designed a novel amphiphilic acceptor **A1** based on the prototypical acceptor 9,10-diphenylanthracene (DPA). Quaternary ammonium groups were introduced at both ends of the DPA chromophore, by reference to the molecular design of aqueous monolayer membranes.^36^–^38^ The DPA unit is modified with amide groups since the formation of dense hydrogen bonding networks would be beneficial for blocking oxygen.^28^,^35^ The long alkyl chain spacer groups locate the amide groups in the hydrophobic interior of the molecular membrane structure, which would facilitate the formation of hydrogen bond networks in water.^39^ While the typical donor Pt(ii) octaethylporphyrin (PtOEP) was too hydrophobic to be stably dispersed in the aqueous self-assemblies of **A1**, anionic donor PtP4COONa co-assembles with cationic **A1**, to which triplet energy was efficiently transferred (Fig. 2). These donor–acceptor co-assemblies showed an efficient TEM-UC emission in deaerated aqueous dispersions, which was largely preserved even in the air-saturated aqueous systems. Control experiments in a water/DMF mixed solvent and with another acceptor **A2** that does not have amide groups showed no UC emission, indicating the important roles that dense molecular packing and intermolecular networks play in blocking oxygen molecules.

## Results and discussion

**Fig. 3:**
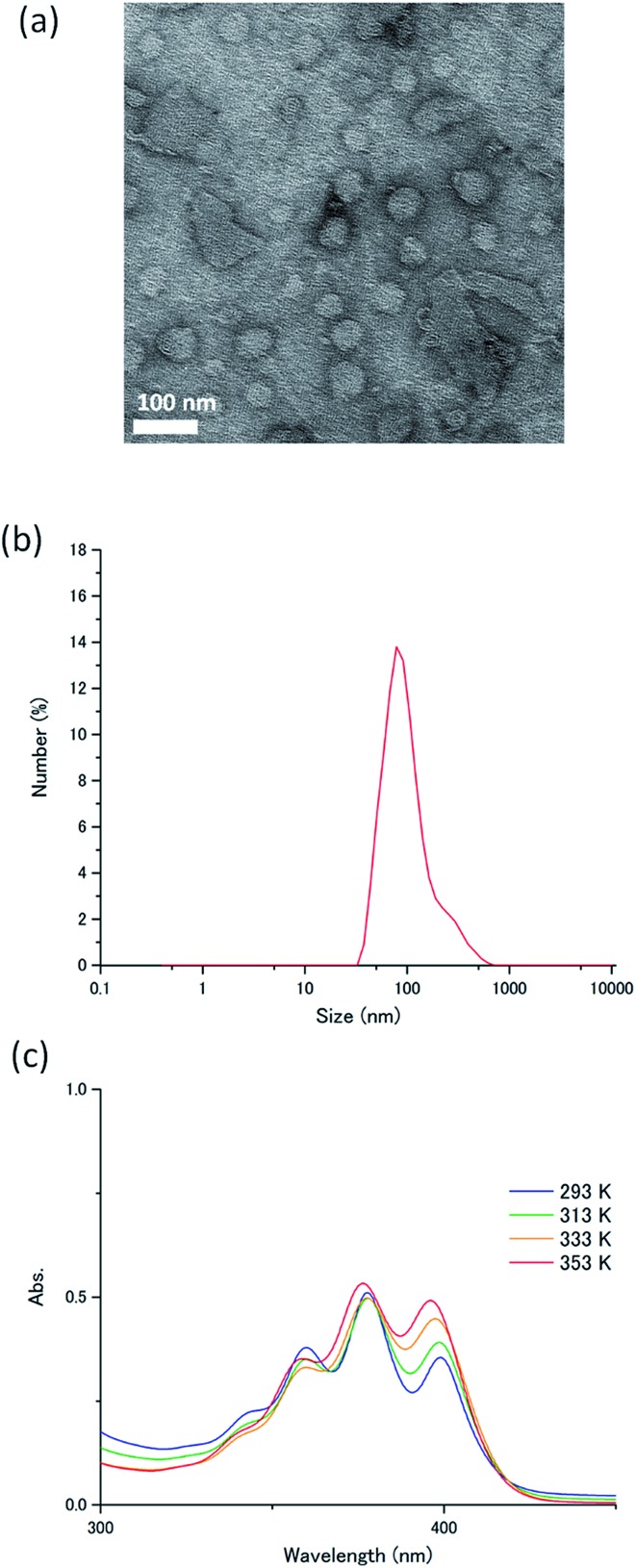
(a) Transmission electron microscopy image of **A1**. The sample was post stained with uranyl acetate. (b) DLS profile of **A1** in water ([**A1**] = 1 mM). (c) Temperature-dependent absorption spectra of **A1** in water ([**A1**] = 1 mM).

The novel acceptor **A1** was synthesized and fully characterized. Its purity was confirmed by ^1^H-NMR measurements and elemental analysis (ESI†). The self-assembly behaviour of **A1** in water was studied using transmission electron microscopy, dynamic light scattering (DLS), IR spectroscopy and temperature-dependent absorption spectroscopy. Transmission electron microscopy conducted for the aqueous dispersion of **A1** exhibited round nanostructures with an average diameter of *ca.* 100 nm (Fig. 3a). This was consistent with the DLS profile of **A1** in water ([**A1**] = 1 mM), which indicated the formation of nanoassemblies with an average size of 110 nm (Fig. 3b). The IR spectrum of **A1** cast film showed a C=O stretching vibration at 1631 cm^–1^ and N–H stretching band at 3319 cm^–1^, which indicate the formation of intermolecular hydrogen bonding (Fig. S1, ESI†).^31^,^40^,^41^ Importantly, these IR bands were maintained for the aqueous dispersion of **A1** (Fig. S1, ESI†). These results clearly confirm that the acceptor amphiphile **A1** forms supramolecular nanoassemblies in water which are stabilized by hydrogen bonding and hydrophobic interactions.

The absorption spectrum of **A1** in water ([**A1**] = 1 mM) exhibited peaks of ^1^L_a_ transitions at 360, 377.5 and 399 nm at 293 K, corresponding to transition dipoles polarized along the short axis of the anthracene moiety.^42^ Upon heating the aqueous dispersion to 363 K, these bands showed small blue shifts to 358.5, 376, and 395.5 nm (Fig. 3c). The observed small spectral shifts indicate the presence of weak interactions between the transition moments, probably because of the steric effects caused by the conformationally twisted benzene groups attached to the 9,10-positions of the anthracene chromophore. The fluorescence spectrum of **A1** in water shows a band at around 440 nm, which doesn't show large overlaps with the donor absorption spectra (Fig. S2, ESI†). A high absolute fluorescence quantum yield of 53% was observed for **A1** in water ([**A1**] = 1 mM).

While the acceptor DPA units are densely arrayed in the **A1** assembly, the conformationally twisted phenyl rings attached to the anthracene moiety seem to suppress the strong interchromophore interactions and concomitant quenching of the fluorescence, as observed in absorption and emission behaviours.

The dense molecular alignment with hydrogen bond networks formed in **A1** assemblies effectively prevent the quenching of the UC emission by oxygen in water. The photoluminescence spectra of **A1** and PtOEP in water ([**A1**] = 1 mM, [PtOEP] = 1 μM) were measured after deaeration by repeated freeze–pump–thaw cycles. Under excitation at 532 nm, an UC emission was clearly observed at around 440 nm (Fig. S3a, ESI†), and the spectral shape matches well with the fluorescence spectrum of **A1** (Fig. S2, ESI†). Very interestingly, this UC emission was found to be largely preserved also in the air-saturated aqueous dispersion (Fig. S3a, ESI†). To understand the role of the intermolecular network structure, we carried out control experiments by synthesizing another new DPA-based amphiphilic acceptor **A2** that does not possess amide groups (Fig. S4, ESI†). The **A2** molecules also form self-assembled nanostructures in water, as evidenced by transmission electron microscopy, DLS and temperature-dependent absorption measurements (Fig. S4, ESI†). The **A2**–PtOEP also showed an UC emission in the deaerated aqueous dispersion ([**A2**] = 1 mM, [PtOEP] = 1 μM), however, this UC emission entirely disappeared in the aerated specimens (Fig. S3b, ESI†). These results indicate that the dense molecular alignment promoted by the developed intermolecular hydrogen bonding networks is crucial to avoid oxygen quenching.

We should note that the UC emission of the **A1**–PtOEP system was weak even in the deaerated dispersion, which is accompanied by the phosphorescence of PtOEP at 650 nm. Upon incubating the aqueous **A1**–PtOEP dispersion for 24 h, precipitation of the red PtOEP aggregates was observed. Apparently, the hydrophobic donor PtOEP is not co-dispersed stably and tends to segregate, resulting in poor donor-to-acceptor TTET efficiency.

**Fig. 4:**
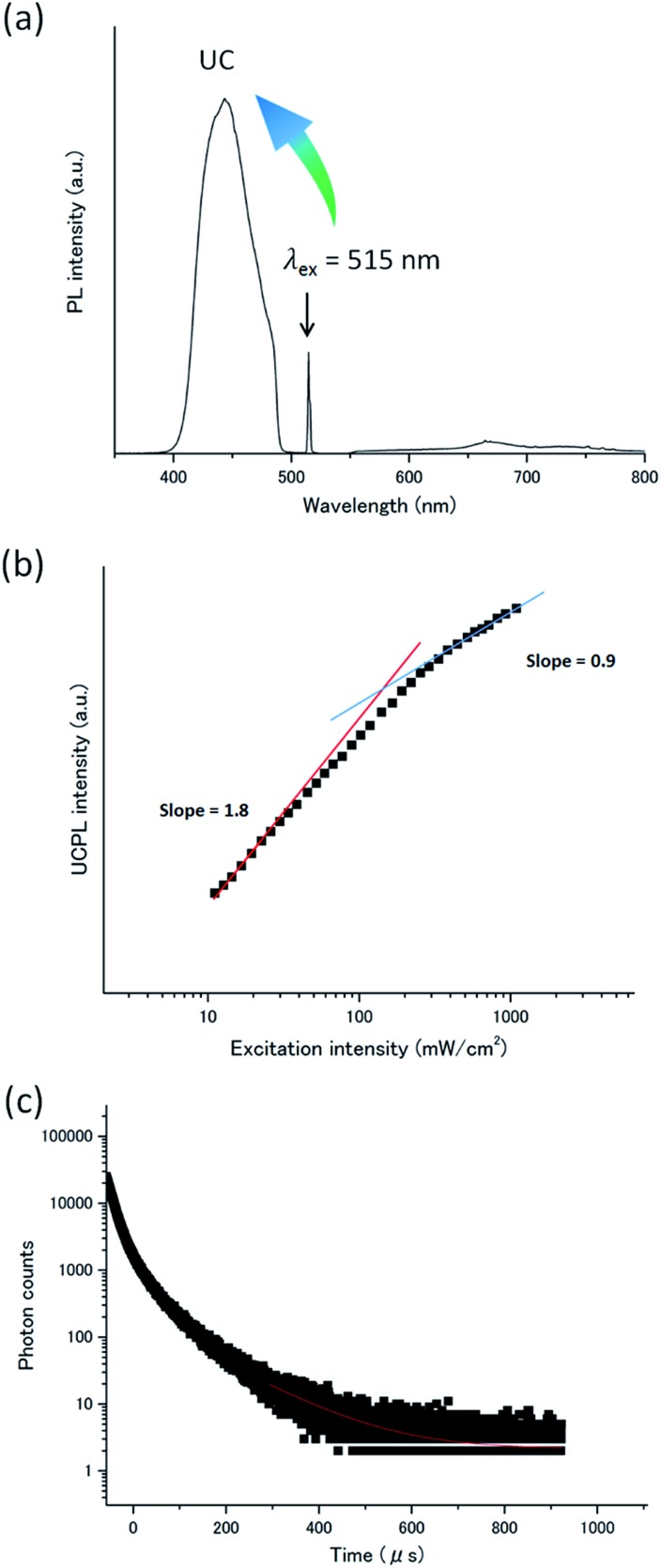
(a) Photoluminescence spectrum of **A1**–PtP4COONa in deaerated water ([**A1**] = 1 mM, [PtP4COONa] = 1 μM, *λ*_ex_ = 515 nm, excitation intensity = 275 mW cm^–2^). Scattered incident laser light was removed by 490 nm short-pass filter (for UC emission) and by 550 nm long-pass filter (for phosphorescence). (b) UC emission intensity at 440 nm of **A1**–PtP4COONa in deaerated water as a function of the excitation intensity ([**A1**] = 1 mM, [PtP4COONa] = 1 μM, *λ*_ex_ = 515 nm). The solid red and blue lines are fitting results with slopes of 1.8 and 0.9 in the low and high power regimes, respectively. (c) UC emission decay at 440 nm of **A1**–PtP4COONa in deaerated water under 531 nm pulsed excitation. The red fitting curve in the tail part of the decay was obtained by considering the relationship of *I*_UC_(*t*) ∝ exp(–*t*/*τ*_UC_) = exp(–2*t*/*τ*_A,T_), where *τ*_UC_ is UC emission lifetime and *τ*_A,T_ is acceptor triplet lifetime.

Meanwhile, by employing the anionic donor PtP4COONa (Fig. 2), the compatibility with the cationic acceptor **A1** was improved. When PtP4COONa was mixed with **A1** in deaerated dispersion at room temperature ([**A1**] = 1 mM, [PtP4COONa] = 1 μM), only donor phosphorescence was observed with almost no UC emission, reflecting that the rigid molecular assembly of **A1** did not accommodate donor molecules. To co-assemble the donor and acceptor, a mixed aqueous dispersion of **A1**–PtP4COONa was heated in a microwave at 393 K for 30 min and incubated at room temperature under stirring for 48 h. The obtained aqueous dispersion was deaerated by repeated freeze–pump–thaw cycles. Interestingly, the obtained dispersion showed a clear UC emission at 440 nm (Fig. 4a). Almost no donor phosphorescence at 670 nm indicates the integration of donor molecules in acceptor assemblies, facilitating efficient donor-to-acceptor TTET. The heating procedure may have disassembled the acceptor nanostructure but it ensured the electrostatic co-assembly between the acceptor and donor.

The TTA-based UC mechanism was confirmed by the excitation intensity dependence and lifetime of the UC emission. The TTA-based UC emission intensity generally shows a quadratic and first-order dependence on the incident intensity in the low- and high-excitation intensity ranges, respectively. A double logarithmic plot for the UC emission intensity of the **A1**–PtP4COONa aqueous dispersion as a function of incident light intensity showed a transition from a slope of *ca.* 2 to 1, providing unequivocal evidence for TTA-based UC (Fig. 4b). The *I*_th_ value of 130 mW cm^–2^ is experimentally determined as the intersection point. The TTA-based mechanism was further supported by the UC emission decay in microsecond time scale characteristics to UC *via* a long-lived triplet state (Fig. 4c). It has been well documented that the TTA-based delayed fluorescence is not a single exponential decay, but its tail part obeys single exponential behavior.^3^,^43^ The acceptor triplet lifetime *τ*_A,T_ was obtained as 238 μs by considering the known relationship of *I*_UC_(*t*) ∝ exp(–2*t*/*τ*_A,T_).^3^,^43^

The triplet diffusion constant for the acceptor arrays *D*_T_ was estimated by using the following relationship,^44^

1 *I*_th_ = (*αΦ*_ET_8π*D*_T_*a*_0_)^–1^(*τ*_A,T_)^–2^,

where *α* is the absorption coefficient at the excitation wavelength in cm^–1^, *Φ*_ET_ is the donor-to-acceptor TTET efficiency (*ca.* 1), and *a*_0_ is the annihilation distance between acceptor triplets (9.1 Å for DPA triplets).^44^ From these parameters, we obtained a fairly high *D*_T_ value of 1.4 × 10^–4^ cm^2^ s^–1^, being higher than the diffusion constant of DPA in a low-viscosity solvent (1.2 × 10^–5^ cm^2^ s^–1^).^44^

**Fig. 5:**
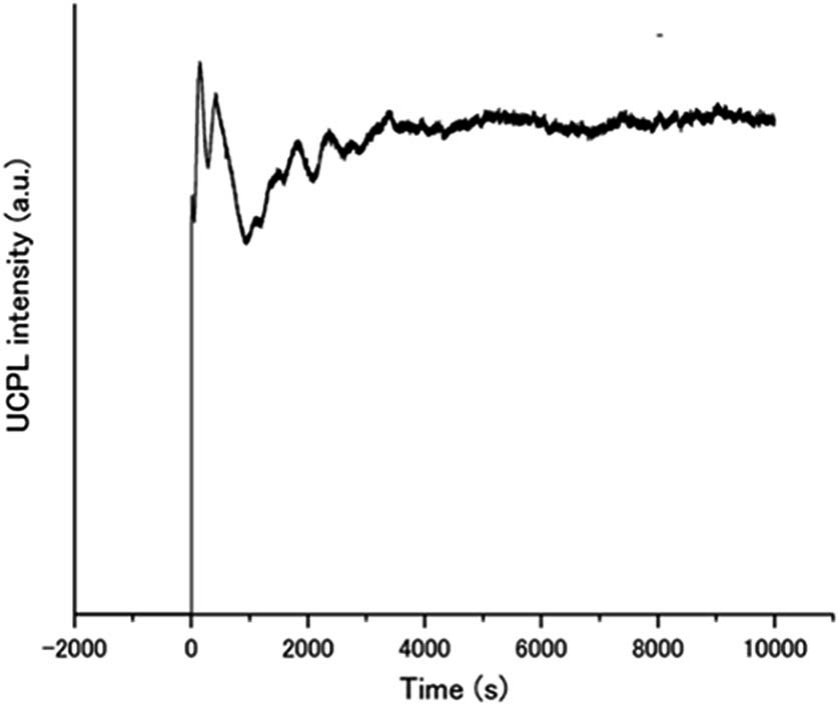
Time dependence of the UC emission intensity of **A1**–PtP4COONa in air-saturated water at 450 nm upon continuous excitation at 515 nm with a laser light intensity of 5 W cm^–2^.

The remarkable air-stability of the aqueous TEM-UC emission was demonstrated by the UC quantum yield and time-dependent UC emission measurements. The UC quantum yield was determined by using an aqueous solution of rhodamine B as a standard. Because the TTA-UC process converts two photons to one photon, the theoretical maximum of the UC quantum yield *Φ*_UC_ is 50%.^10^,^13^,^45^ Meanwhile, in many reports this value is multiplied with 2 to set the maximum conversion efficiency as 100%. To avoid the confusion between these different definitions, the UC quantum yield is written as *Φ*′_UC_ (= 2*Φ*_UC_) when the maximum efficiency is standardized to be 100%. A high *Φ*′_UC_ value of 13% was obtained for **A1**–PtP4COONa in deaerated water (Fig. S5, ESI†). Note that the fluorescence lifetime of **A1** was not almost affected by the donor PtP4COONa, indicating the absence of acceptor-to-donor singlet–singlet back energy transfer (Fig. S6, ESI†). Significantly, even in the presence of dissolved oxygen, more than half of the *Φ*′_UC_ value was maintained (7.0%). This quantum yield is comparable to those of previous highly efficient molecular-diffusion-based UC systems in aerated conditions.^21^,^22^ The time-dependent UC emission of **A1**–PtP4COONa in the air-saturated aqueous dispersion maintained peak intensity right after starting the laser irradiation for more than 10 000 seconds (Fig. 5). It has been reported that the UC emission becomes stronger with time when oxygen molecules are consumed by photochemical reactions.^5^,^28^ The absence of such a gradual increase in emission intensity indicates the intrinsic oxygen barrier ability of the aqueous molecular assembly system.

**Fig. 6:**
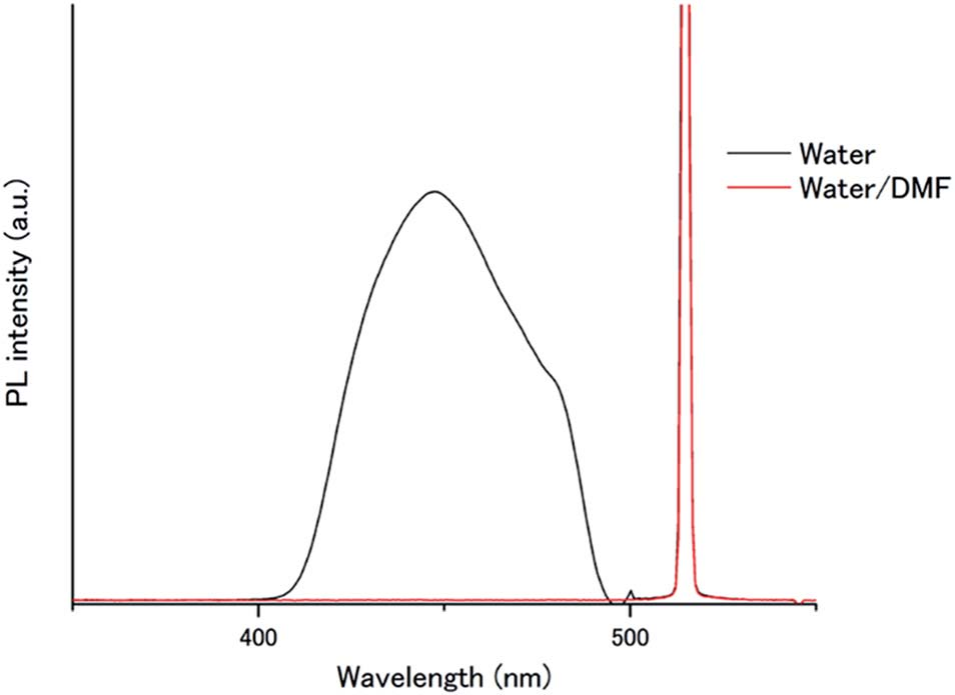
Photoluminescence spectra of **A1**–PtP4COONa ([**A1**] = 1 mM, [PtP4COONa] = 1 μM) in aerated water (black) and water/DMF (red) (*λ*_ex_ = 515 nm, excitation intensity = 1.3 W cm^–2^). Scattered incident light was removed by a 490 nm short-pass filter.

To understand this air-stable behaviour, we carried out control experiments by employing a water/DMF (1 : 1 in volume) mixed solvent instead of pure water for the **A1**–PtP4COONa pair to weaken the hydrophobic interactions and eventually break the hydrogen bonding networks. In contrast to the temperature-dependent shifts observed in the absorption spectra of **A1** by supramolecular assembly/disassembly (Fig. 3c), the absorption peaks of **A1** at 357.5, 376, 396 nm did not show any shift between 298 K and 363 K in water/DMF, indicating the absence of interchromophore close-contact assembly in water/DMF (Fig. S7†). We compared the photoluminescence spectra of the **A1**–PtP4COONa pair in air-saturated water and water/DMF (Fig. 6). Very interestingly, the UC emission, observed in aerated water, was completely quenched in aerated water/DMF. This clear difference supports the principal role of dense molecular alignment of **A1** promoted by hydrogen bonding networks in monolayer membranes in avoiding oxygen quenching. Furthermore, the observed air stability of **A1**–PtP4COONa in pure water is in stark contrast to the control system without hydrogen bond networks, **A2**–PtP4COONa, for which the UC emission was completely quenched in aerated water similar to the case of **A2**–PtOEP. These results clearly demonstrate the proper amphiphilic molecular design of acceptor chromophores with dense intermolecular networks such as hydrogen bonds to achieve the air-stable TEM-UC aqueous system.

## Conclusions

A supramolecular TEM-UC system showing an air-stable UC emission in an aqueous environment was developed for the first time. The rational design guideline to achieve this goal is demonstrated; the penetration of oxygen molecules is effectively blocked by the dense intermolecular networks driven by hydrogen bonding interactions in the hydrophobic environment of the acceptor self-assemblies. The stabilized acceptor triplets, sensitized by the co-assembled donor, migrate in the acceptor arrays and annihilate to produce efficient UC emission. The chromophores self-assembled in the hydrophobic interior with developed hydrogen bond networks of aqueous assembly systems thus are less influenced by dissolved molecular oxygen, which provides a new perspective in the design of photochemical or photophysical functions that require the interplay of reactive photo-generated species as observed in natural thylakoid membranes.

## Supplementary Material

Supplementary information
