# Supplementary material for: Triplet energy migration-based photon upconversion by amphiphilic molecular assemblies in aerated water
Source: Chem Sci. 2016 Apr 18;7(8):5224–9. doi: 10.1039/c6sc01047d (PMC6020542; doi:10.1039/c6sc01047d)
Supplement: Supplementary file 1 [file SC-007-C6SC01047D-s001.pdf]

Electronic Supplementary Material (ESI) for Chemical Science.
This journal is © The Royal Society of Chemistry 2016

Supporting Information for

Triplet energy migration-based photon upconversion by amphiphilic molecular assemblies in aerated water

Hironori Kouno,[a] Taku Ogawa,[a] Shogo Amemori,[a] Prasenjit Mahato,[a] Nobuhiro Yanai,*[a,b] and Nobuo Kimizuka*[a]

[a]Department of Chemistry and Biochemistry, Graduate School of Engineering, Center for Molecular Systems (CMS), Kyushu University, 744 Moto-oka, Nishi-ku, Fukuoka 819-0395, Japan.

[b]PRESTO, JST, Honcho 4-1-8, Kawaguchi, Saitama 332-0012, Japan.

E-mail: yanai@mail.cstm.kyushu-u.ac.jp; n-kimi@mail.cstm.kyushu-u.ac.jp.

Materials.

All reagents and solvents for synthesis were used as received without further purification. Pt (II) octaethylporphyrin (**PtOEP**) was purchased from Aldrich and used as received. PtP4COONa was synthesized according to the reported procedure.[1] ^{1}H NMR (300 MHz, $CDCl_3$): δ (ppm) = 1.20 (d, 24H), 2.78 (quintet, 4H), 7.36 (d, 4H), 7.50 (t, 2H), 8.66 (d, 4H), 8.75 (s, 4H), 8.77 (d, 4H).

Synthesis of Acceptor 1.

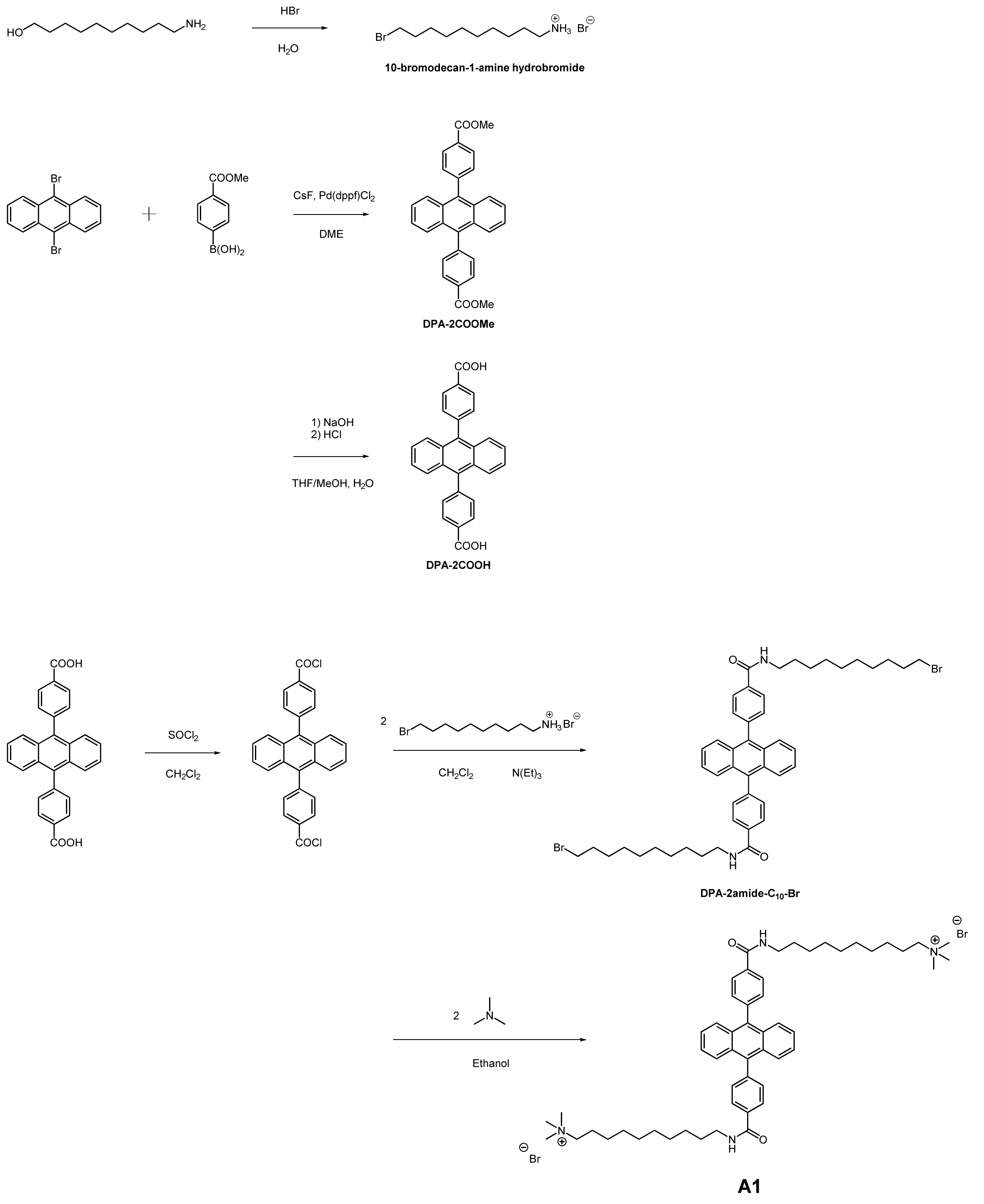

Synthesis of 10-bromodecane-1-ammonium bromide.[2] 5.1 ml of hydrobromic acid (48 %) was placed in 100 ml flask and cooled by using ice bath, to which 500 mg (2.9 mmol) of 10-aminodecane-1-ol was slowly added. The suspension was refluxed for 6 h under Ar. The solvent was removed under reduced pressure and the product was purified by recrystallization in toluene/ethanol (50/1) to give a colorless solid 10-bromodecane-1-ammonium bromide (yield : 74.2%).

^{1}H NMR (300 MHz, $CDCl_3$): δ (ppm) = 1.25-1.47 (m, 12H), 1.77-1.85 (m, 4H), 2.97-3.10 (m, 2H), 3.41 (t, 2H), 8.05 (s, 3H).

Synthesis of DPA-2COOMe. [3] A mixture of 2.50 g (14 mmol) (4-(methoxycarbonyl)phenyl)boronic acid, 1.50 g (4.5 mmol) 9,10-dibromo anthracene, 2.47 g (16.3 mmol) CsF and 110 mg (0.135 mmol) [1,1'-Bis(diphenylphosphino)ferrocene]palladium(II) dichloride dichloromethane Adduct were placed in a 300 ml flask under Ar, and 75 ml of degassed 1,2-dimethoxyethane were added. After refluxing under Ar for 50 h, the solvent was removed to give a yellow residue. This obtained solid was suspended in 35 ml water and extracted with 100 ml of $CHCl_3$. After drying the organic phase over Na_2SO_4 and removing the solvent, the product was purified by column chromatography ($CHCl_3$) over silica gel to yield of a yellow powder (yield: 47.1%).

^{1}H NMR (300 MHz, $CDCl_3$): δ (ppm) = 4.03 (s, 6H), 7.56 (dd, 4H), 7.63 (dd, 8H), 8.31 (dd, 4H).

Synthesis of DPA-2COOH.[3] To a suspension of 410 mg (9.18 mmol) of dimethyl 4,4-(anthracene-9,10-diyl)dibenzoate in 150 ml 1:1 mixture of THF/MeOH, 30 ml of a 2 M KOH aqueous solution was added. The mixture was heated to 40 ºC for 1 day. The mixture formed by acidification with aqueous HCl (2 M) was collected by filtration, washed several times with water, yielding 204 mg (yield: 53.1 %) of a pale yellow solid.

^{1}H NMR (300 MHz, DMSO-d_6): δ (ppm) = 7.44-7.49 (m, 4H), 7.51-7.58 (m, 4H), 7.64 (dd, 4H), 8.22 (dd, 4H).

Synthesis of DPA-2amide-C_{10}-Br.[4,5] 150 mg (0.36 mmol) of 4,4-(anthracene-9,10-diyl)dibenzoic acid was placed in 50 ml flask, and 10 ml of dehydrated benzene was added under Ar. Then, 1 ml of thionyl chloride were added to the suspension and refluxed for 5 h with catalyst quantity of dehydrated DMF. After removing the solvent and excess thionyl chloride under reduced pressure, residual yellow solid was dispersed into 15 ml of distilled dichloromethane, and this solution was added dropwise to 249 mg (0.79 mmol) of 10-bromodecane-1-ammonium bromide dissolved in 300 ml of dehydrated dichloromethane and 350 μl of trimethylamine under Ar. This mixture was stirred at room temperature for 1 day. After the reaction, the solution was washed several times with diluted hydrochloric acid aqueous solution, Na_2SO_4 aqueous solution, water with dichloromethane. Evaporation of the organic layer under reduced pressure and column chromatography with chloroform/methanol (30/1) over silica gel yielded 20 mg (yield: 5.4%) of a colorless solid.

^{1}H NMR (300 MHz, DMF-d_7): δ (ppm) = 1.30-1.45 (m, 24H), 1.50-1.58 (m, 4H), 1.70 (quin, 4H), 1.90 (quin, 4H), 3.42 (t, 4H), 3.55 (quin, 4H), 6.27 (t, 2H), 7.34 (q, 4H), 7.58 (dd, 4H), 7.61 (quin, 4H), 8.06 (dd, 4H).

Synthesis of A1.[6,7] 20 mg (0.023 mmol) of DPA-2amide-C_{10}-Br was dissolved in 10 ml of trimethylamine ethanol solution. The solution was heated by microwave at 80 °C for 14 h. After the reaction, the solution was cooled to room temperature. The precipitated colorless solid product was purified by filtration to provide **A1** (yield: 52.8%). ^{1}H NMR (300 MHz, DMF-d_7): δ (ppm) = 1.30-1.50 (m, 8H), 1.65-1.75 (m, 4H), 1.85-1.95 (m, 4H), 3.49-3.51. (m, 8H), 7.51-7.55 (m, 4H), 7.62-7.66 (m, 4H), 8.32 (dd, 4H), 8.76 (t, 2H).

Elemental analysis for $C_{54}H_{76}N_4O_2Br_2$: calculated (%) H 7.87 C 66.66 N 5.76; found (%) H 7.62 C 66.41 N 5.62.

Synthesis of Acceptor 2.

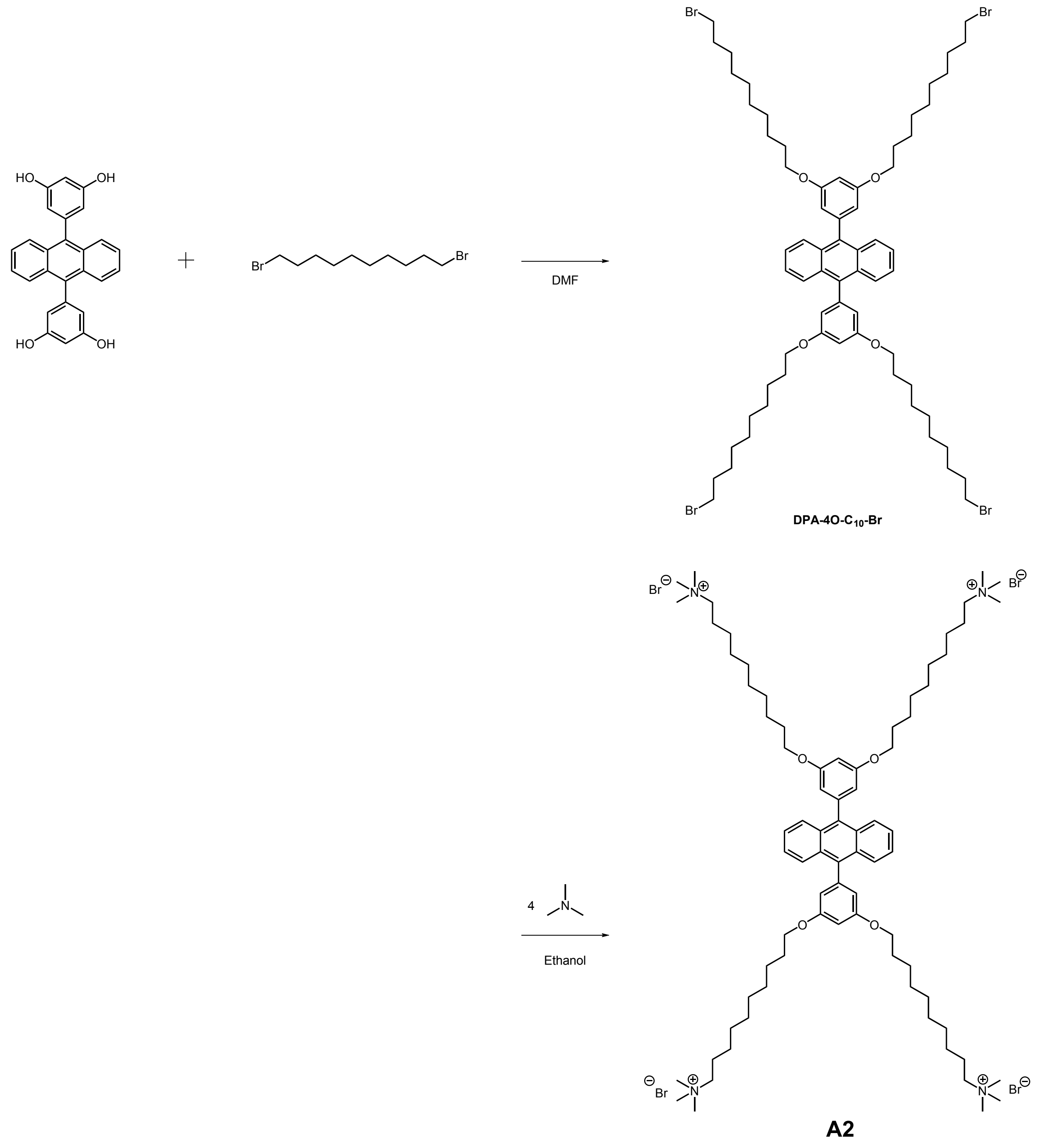

Synthesis of DPA-4O-C_{10}-Br.[8] 0.78 g (2.0 mmol), 1,10-dibromodecane, 6.0 g (20 mmol), and Potassium carbonate 1.38 g (10 mmol) were placed in 100 ml flask under N_2, and 60 ml of dehydrated DMF was added. The solution was stirred at room temperature for 24 h. After the reaction, the solution was washed several times with hexane/dichloromethane mixture (1/5) and water, dried over anhydrous Na_2CO_3. The solvent was removed under reduced pressure, and the product was purified by recrystallization in ethyl acetate and hexane (1/49) to give needle crystals DPA-4O-C_{10}-Br (yield: 22.6%).
^{1}H NMR (300 MHz, $CDCl_3$): δ (ppm) = 1.20-1.50 (m, 48H), 1.81 (sep, 16H), 3.39 (t, 8H), 3.97 (t, 8H), 6.61 (d, 4H), 6.63 (t, 2H), 7.33 (dd, 4H), 7.78 (dd, 4H).

Synthesis of A2.[6,7] 413 mg (0.33 mmol) of DPA-4O-C_{10}-Br was dissolved in 10 ml of trimethylamine ethanol solution. The solution was heated by microwave at 80 °C for 14 h. After the reaction, the solution was cooled to room temperature, and the precipitated colorless solid product was purified by filtration to provide **A2** (yield: 24.6%).
^{1}H NMR (300 MHz, DMF-d_7): δ (ppm) = 1.30-1.50 (m, 48H), 1.80-1.90 (m, 16H), 3.35 (s, 36H), 4.13 (t, 8H), 6.66 (d, 4H), 6.82 (t, 2H), 7.51 (dd, 4H), 7.78 (dd, 4H).
Elemental analysis for $C_{78}H_{130}N_4O_4Br_4$: calculated (%) H 9.96 C 74.58 N 5.32; found (%) H 9.93 C 74.40 N 5.25.

Characterizations.

^{1}H NMR (300 MHz) spectra were measured on a Bruker DRX-spectrometer using TMS as the internal standard. Elemental analysis was conducted at the Elemental Analysis Center, Kyushu University. Absorption spectra were recorded on a JASCO V-670 spectrophotometer. Fluorescence spectra were measured by using a PerkinElmer LS 55 fluorescence spectrometer. Time-resolved photoluminescence lifetime measurements were carried out by using a time-correlated single photon counting lifetime spectroscopy system, HAMAMATSU Quantaurus-Tau C11567-01. Upconverted emission spectra were recorded on a Hamamatsu Photonics PMA-12 with the excitation source using an external, adjustable semiconductor laser (532 nm) and Argon laser GLG3100 (515 nm). The absolute quantum yield was measured in an integrating sphere using a Hamamatsu Photonics absolute quantum yield measurement system. The UC sample preparation was carried out by using Biotage Initiator. Transmission electron microscopy images were measured by using JOEL JEM-2010. Dynamic light scattering measurements were carried out by using Malvern Nano-ZS ZEN3600.

Determination of TTA-UC quantum yield.

The upconverted luminescence quantum efficiency was determined relative to a standard according to the following equation,

$$\Phi_{UC} = \Phi_{std}\left(\frac{A_{std}}{A_{UC}}\right)\left(\frac{I_{UC}}{I_{std}}\right)\left(\frac{\eta_{UC}}{\eta_{std}}\right)^2 = 0.5\,\Phi_{UC}' \qquad \text{(S1)}$$

where Φ, A, I and η represent the quantum yield, absorbance at 385 nm, integrated photoluminescence spectral profile, and a refractive index of the solvent The subscripts *UC* and *std* denote the parameters of the upconversion and standard systems. The quantum efficiency of **A1** was determined relative to a standard, Rhodamine B in water (Φ = 0.25). Note that the theoretical maximum of Φ_{UC}' is standardized to be 1 (100%).

Supporting figures and table:

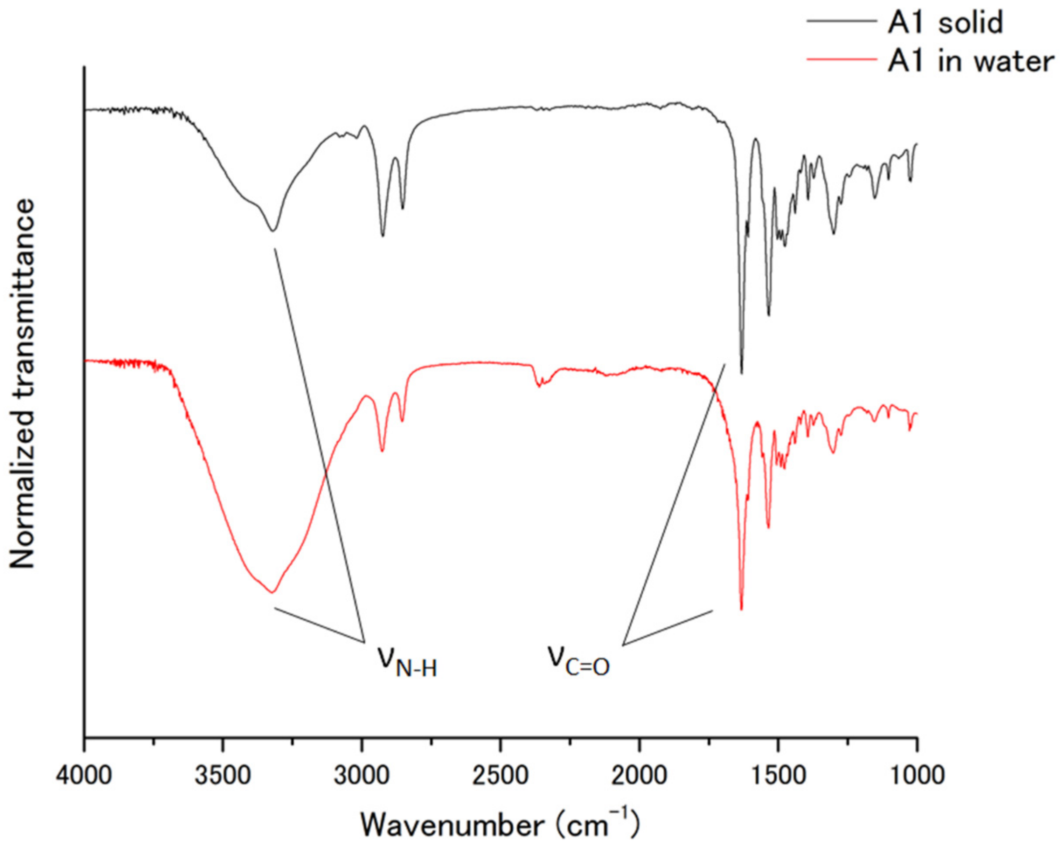

Figure S1. IR spectrum of **A1** in the solid state (black) and in the aqueous dispersion state ([**A1**] = 100 mM). High concentration of **A1** was necessary to observe the IR peaks due to the low sensitivity of IR measurements. The **A1** molecules were nicely dispersed in water without precipitation even in this high concentration condition.

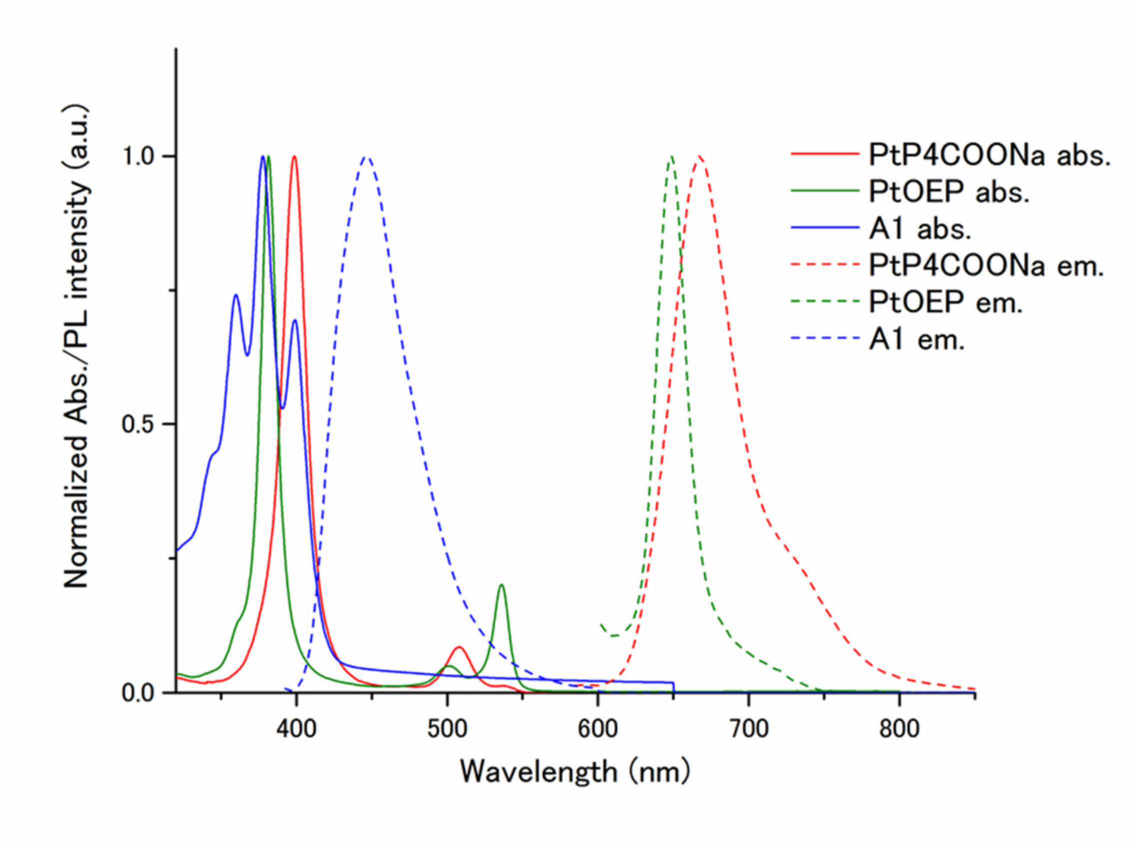

Figure S2. Normalized absorption (solid lines) and emission (dashed lines) spectra of **A1** (blue, λ_{ex} = 375 nm, 1 mM) and PtP4COONa (red, λ_{ex} = 510 nm, 1 μM) in water, and PtOEP in chloroform (green, λ_{ex} = 532 nm, 1 μM) at room temperature.

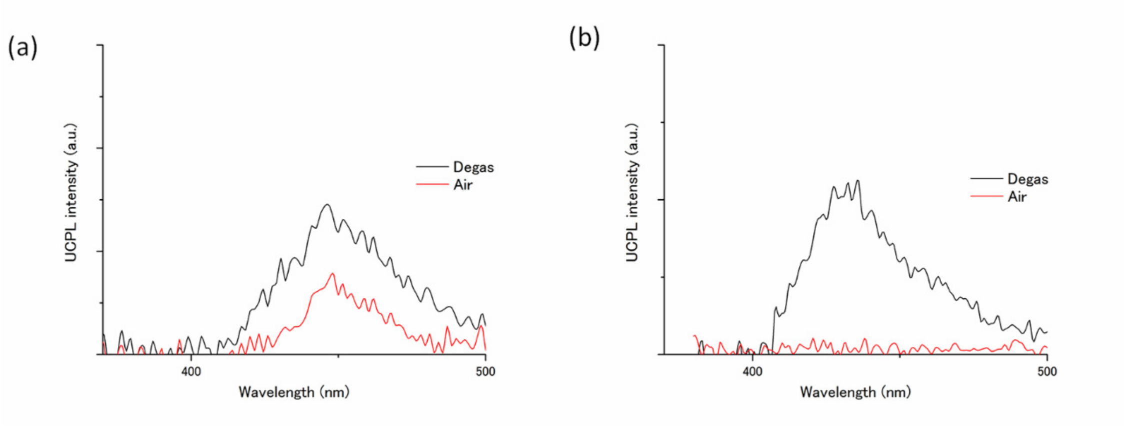

Figure S3. Upconvertd photoluminescence (UCPL) spectra of (a) **A1**-PtOEP (b) **A2**-PtOEP in water ([**A1**] = [**A2**] = 1 mM, [PtOEP] = 1 μM) in the absence (black) and presence (red) of dissolved air (oxygen) under excitation at 532 nm. Scattered incident laser light was removed by a notch filter (532 nm).

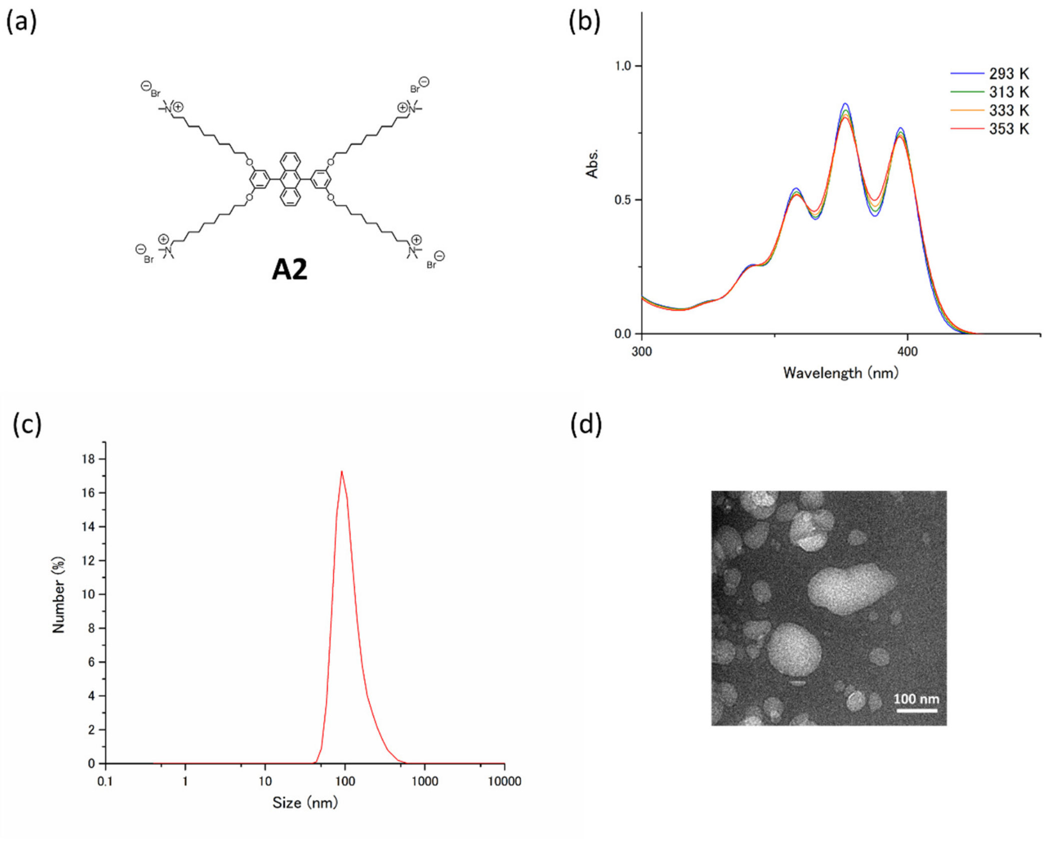

Figure S4. (a) Chemical structure of **A2**. (b) Temperature-dependent absorption spectra of **A2** in water ([**A2**] = 1 mM). (c) DLS profile of **A2** in water ([**A2**] = 1 mM). (d) Transmission electron microscopy image of **A2**. The sample was post stained by uranyl acetate.

Absorption spectrum of **A2** in water exhibited peaks at 358, 376, 396.5 nm at 293 K, which showed small blue shifts to 357, 375, 395.5 nm by heating to 353 K. The DLS profile of **A2** in water indicates the formation of nanoassemblies with an average size of 103.6 nm. The transmission electron microscopy image of **A2** exhibited round nanostructures. These results confirm that the hydrophobic interactions allow **A2** to form supramolecular nanoassemblies in water.

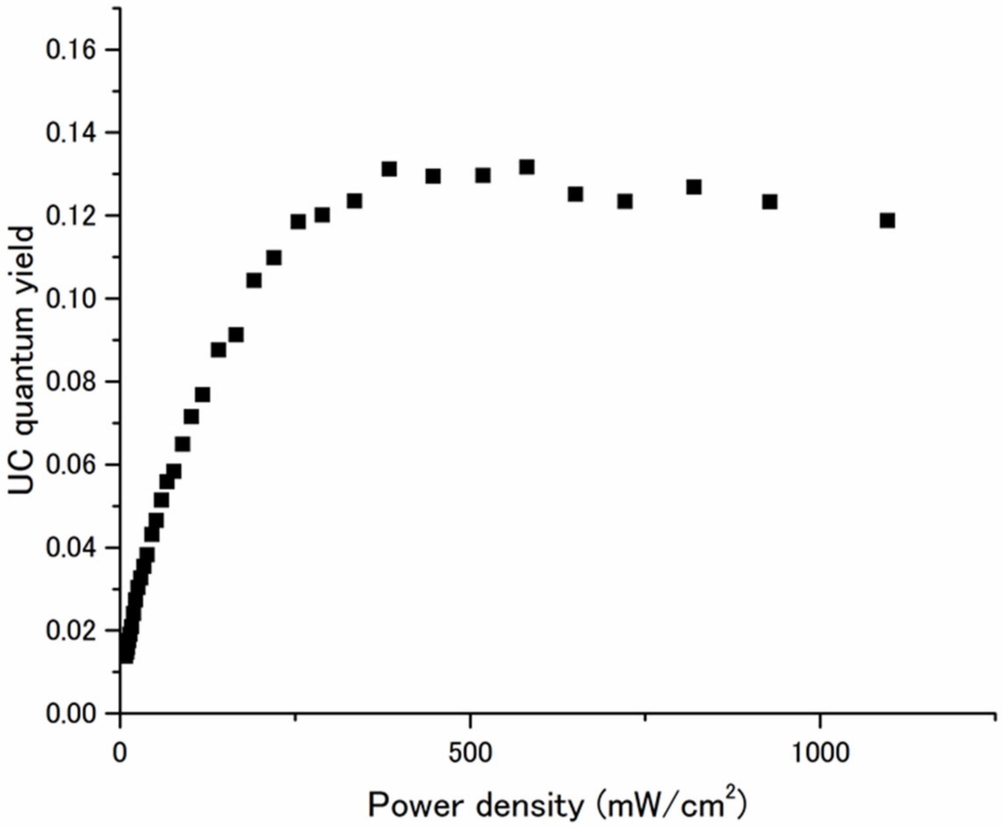

Figure S5. Dependence of UC quantum yield Φ_{UC}' on the excitation intensity of **A1**-PtP4COONa in deaerated water at room temperature ([**A1**] = 1 mM, [PtP4COONa] = 1 μM, λ_{ex} = 515 nm).

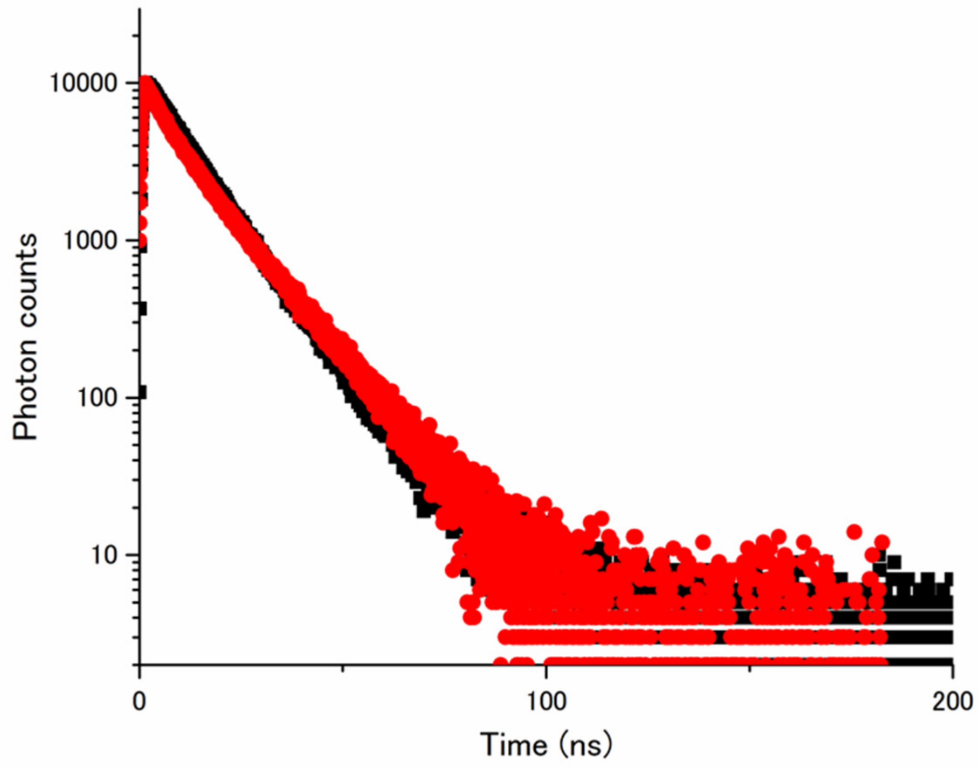

Figure S6. Fluorescence decays at 450 nm for **A1** (black) and **A1**-PtP4COONa (red) in air-saturated water under pulsed excitation at 365 nm ([**A1**] = 1 mM, [PtP4COONa] = 1 μM).

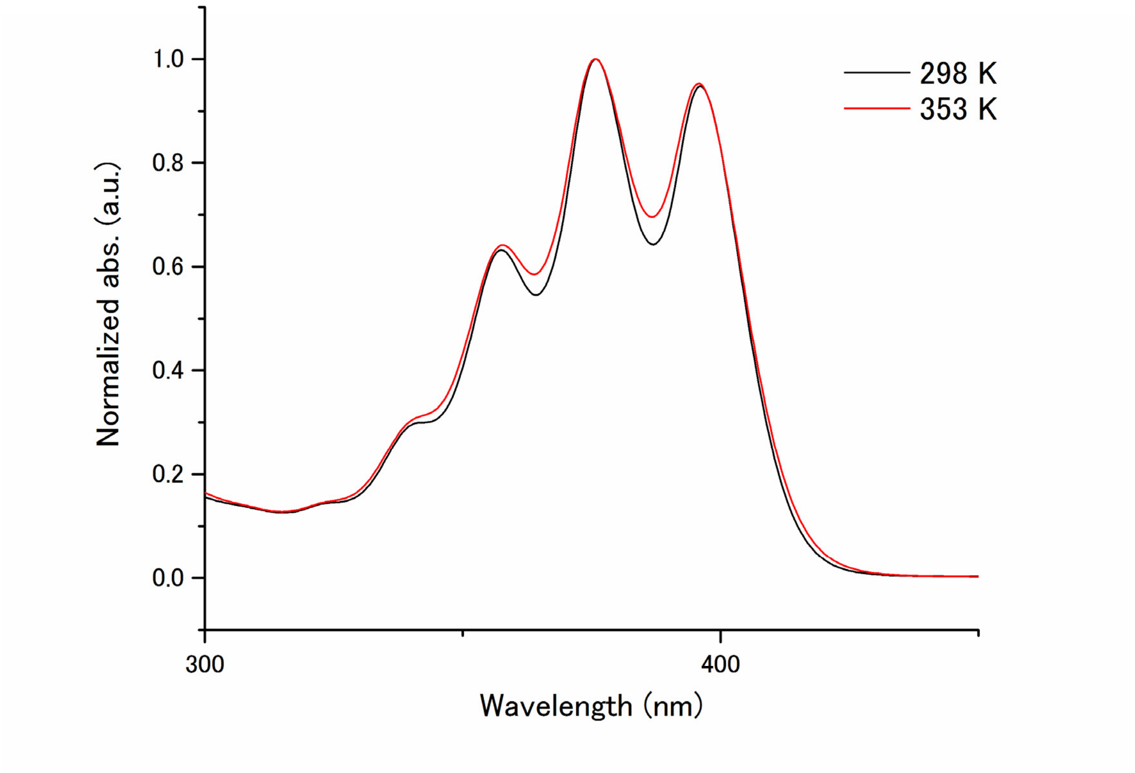

Figure S7. Absorption spectra of **A1**-PtP4COONa ([**A1**] = 1 mM, [PtP4COONa] = 1 μM) at 298 K (black) and 353 K (red) in DMF/water (1:1 in volume).

Supporting references:

1. D. Feng, Z.-Y. Gu, J.-R. Li, H.-L. Jiang, Z. Wei and H.-C. Zhou, *Angew. Chem., Int. Ed.*, 2012, **51**, 10307–10310.
2. R. Sauer, A. Turshatov, S. Baluschev and K. Landfester, *Macromolecules*, 2012, **45**, 3787–3796.
3. D. Shi, Y. Ren, H. Jiang, J. Lu and X. Cheng, *Dalt. Trans.*, 2012, 484–491.
4. D. N. Reddy and E. N. Prabhakaran, *J. Org. Chem.*, 2011, **76**, 680–683.
5. A. Brown, K. M. Mullen, J. Ryu, M. J. Chmielewski, S. M. Santos, V. Felix, A. L. Thompson, J. E. Warren, S. I. Pascu and P. D. Beer, *J. Am. Chem. Soc.*, 2009, **131**, 4937–4952.
6. S. Chavda, G. S. Coumbarides, M. Dingjan, J. Eames and A. Zarbakhsh, *J. Label. Compd. Radiopharm.*, 2006, **49**, 147–151.
7. S. De, V. K. Aswal and S. Ramakrishnan, *Langmuir*, 2010, **26**, 17882–17889.
8. J. Nithyanandhan and N. Jayaraman, *J. Org. Chem.*, 2002, **67**, 6282–6285.
